# Reciprocal sensitivity of diffuse large B-cell lymphoma cells to Bcl-2 inhibitors BIRD-2 versus venetoclax

**DOI:** 10.18632/oncotarget.22898

**Published:** 2017-12-04

**Authors:** Tamara Vervloessem, Haidar Akl, Thomas Tousseyn, Humbert De Smedt, Jan B. Parys, Geert Bultynck

**Affiliations:** ^1^ KU Leuven, Laboratory of Molecular and Cellular Signaling, Department of Cellular and Molecular Medicine & Leuven Kanker Instituut (LKI), Leuven, Belgium; ^2^ Current/Present address: Lebanese University, Department of Biology, Hadath, Lebanon; ^3^ KU Leuven, Translational Cell & Tissue Research, Department of Imaging & Pathology, Leuven, Belgium

**Keywords:** apoptosis, anti-apoptotic Bcl-2, B-cell lymphoma, venetoclax, BIRD-2

## Abstract

Bcl-2 is often upregulated in cancers to neutralize the BH3-only protein Bim at the mitochondria. BH3 mimetics (e.g. ABT-199 (venetoclax)) kill cancer cells by targeting Bcl-2’s hydrophobic cleft and disrupting Bcl-2/Bim complexes. Some cancers with elevated Bcl-2 display poor responses towards BH3 mimetics, suggesting an additional function for anti-apoptotic Bcl-2 in these cancers. Indeed, Bcl-2 via its BH4 domain prevents cytotoxic Ca^2+^ release from the endoplasmic reticulum (ER) by directly inhibiting the inositol 1,4,5-trisphosphate receptor (IP_3_R). The cell-permeable Bcl-2/IP_3_R disruptor-2 (BIRD-2) peptide can kill these Bcl-2-dependent cancers by targeting Bcl-2’s BH4 domain, unleashing pro-apoptotic Ca^2+^-release events. We compared eight “primed to death” diffuse large B-cell lymphoma cell lines (DLBCL) for their apoptotic sensitivity towards BIRD-2 and venetoclax. By determining their IC_50_ using cytometric cell-death analysis, we discovered a reciprocal sensitivity towards venetoclax versus BIRD-2. Using immunoblotting, we quantified the expression levels of IP_3_R2 and Bim in DLBCL cell lysates, revealing that BIRD-2 sensitivity correlated with IP_3_R2 levels but not with Bim levels. Moreover, the requirement of intracellular Ca^2+^ for BIRD-2- *versus* venetoclax-induced cell death was different. Indeed, BAPTA-AM suppressed BIRD-2-induced cell death, but promoted venetoclax-induced cell death in DLBCL cells. Finally, compared to single-agent treatments, combining BIRD-2 with venetoclax synergistically enhanced cell-death induction, correlating with a Ca^2+^-dependent upregulation of Bim after BIRD-2 treatment. Our findings suggest that some cancer cells require Bcl-2 proteins at the mitochondria, preventing Bax activation via its hydrophobic cleft, while others require Bcl-2 proteins at the ER, preventing cytotoxic Ca^2+^-signaling events via its BH4 domain.

## INTRODUCTION

Diffuse large B-cell lymphoma (DLBCL) and chronic lymphocytic leukemia (CLL) are amongst the most prevalent lymphoproliferative malignancies, characterized by an aggressive or indolent clinical behavior, respectively. A combination of chemo-, radio- and immunotherapy are currently used to treat patients suffering from non-Hodgkin lymphoma. Although the standard therapy varies according to the lymphoma subtype, often a cocktail of cytotoxic drugs in combination with an anti-CD20 monoclonal antibody (like rituximab^®^) is used in DLBCL. Still, a significant proportion of patients (29% of CD20-positive B-cell malignancies [1]) experiences side effects or relapse after rituximab^®^ treatment due to tumorescape mechanisms like downregulation of CD20 protein expression [2]. Another important survival strategy of these B-cell malignancies is their upregulation of anti-apoptotic Bcl-2 proteins. Indeed, cancer cells are addicted to Bcl-2 for their survival due to their continuous and permanent ongoing apoptotic signaling [3]. Bcl-2 is the founding member of the Bcl-2 family. Along with Bcl-XL, Bcl-W, Bfl-1 and Mcl-1 it belongs to the anti-apoptotic members. These proteins are composed of four Bcl-2 homology (BH) domains. On the opposite side, the pro-apoptotic family members also consist out of 4 BH domains (like Bax and Bak) or have only one (the BH3-only proteins; like Bim) [4]. For many years it is known that anti-apoptotic Bcl-2 via its hydrophobic cleft, formed by the BH1-3, scaffolds and neutralizes pro-apoptotic members of the Bcl-2 family, including the executioner proteins, Bax and Bak, and the BH3-only proteins, like Bim [5]. Additionally, the BH4 domain of Bcl-2 prevented Bax activation by binding to its inactive conformation [6]. This situation where Bcl-2 is loaded with pro-apoptotic family members has been referred to as “primed to death” [7]. Insights into the binding of Bcl-2 with the pro-apoptotic members have spurred the development of the BH3 mimetics. These molecules mimic the BH3 domain of Bad, a sensitizer BH3-only protein thereby disrupting Bcl-2/Bim complexes and releasing Bim from the hydrophobic cleft of Bcl-2, which then results in Bax/Bak-mediated apoptosis in cancer cells but not in healthy cells [8, 9]. Nowadays, a Bcl-2-selective BH3-mimetic compound, ABT-199 (venetoclax) established by AbbVie is currently implemented to treat 17p-deleted CLL patients [10] without affecting platelet survival [11–13]. Unfortunately, some Bcl-2-dependent cancer cells are insensitive to BH3-mimetic compounds [11], likely due to low levels of Bim and/or of Bax/Bak. As such, at the level of the mitochondria, these cells are poorly “primed to death”. Moreover, patients suffering from these poorly-primed cancers also display poor responses to conventional chemotherapeutic drugs and thus seem hard to treat [14].

Interestingly, for treating cancer cells with upregulated Bcl-2 levels, which display reduced responses to chemotherapy/BH3 mimetics, Bcl-2’s function may be used in an alternative way, namely at the endoplasmic reticulum (ER), the main intracellular Ca^2+^-storage organelle [15, 16]. A major ER Ca^2+^-release pathway is formed by the inositol 1,4,5-trisphosphate (IP_3_) receptor (IP_3_R), which impacts several cancer hallmarks, including cell death and survival [17]. During recent years, it became clear that an important part of Bcl-2’s anti-apoptotic properties is due to direct targeting and inhibition of IP_3_Rs [18–20]. Bcl-2/IP_3_R complex formation is dependent on Bcl-2’s BH4 domain, which targets the central, modulatory domain of all three IP_3_R isoforms [21–23]. Importantly, Bcl-2’s hydrophobic cleft is dispensable for IP_3_R-complex formation and IP_3_R inhibition [24]. Consistent with this, venetoclax does neither interfere with the binding of Bcl-2 to IP_3_Rs nor alleviate the inhibition of IP_3_R-mediated Ca^2+^ release by Bcl-2 [24, 25]. Also, a peptide tool comprising the Bcl-2-binding site on the IP_3_R (aa 1389-1408 of mouse IP_3_R1 in which Asp^1403^Asp^1404^ residues were changed into two Ala residues) was developed, called Bcl-2/IP_3_R disruptor-2 (BIRD-2) [26]. BIRD-2 targets the BH4 domain of Bcl-2, thereby disrupting Bcl-2/IP_3_R complexes and thus abolishing Bcl-2’s inhibitory action on the IP_3_R channel, but not Bcl-2/Bim complexes [18]. BIRD-2 by itself is sufficient to kill Bcl-2-dependent CLL and DLBCL cancer cells by provoking spontaneous, pro-apoptotic Ca^2+^ signals [26, 27]. Importantly, BIRD-2 does not cause a general cytotoxicity. Several cell types were found to be very resistant to BIRD-2, e.g. peripheral mononuclear blood cells [26], certain types of DLBCL cells [27], non-malignant cell lines, like WEHI7.2 T cells that express low endogenous Bcl-2 levels [26], and platelets (unpublished data). In addition, the cell-death properties of BIRD-2, which are in lymphoid malignancies accompanied by Bax and caspase-3 activation, also provoked a marked decrease in *in vivo* tumor growth in xenografted mouse models [28]. Remarkably, in these lymphoma cell lines susceptibility to BIRD-2-induced Ca^2+^ release and cell death correlated with the expression level of IP_3_R2. IP_3_R2 is the isoform with the highest sensitivity towards its ligand, IP_3_ [29]. Among DLBCL cancer cells, SU-DHL-4 cells displayed the highest IP_3_R2 level and highest BIRD-2 sensitivity, while OCI-LY-1 displayed the lowest IP_3_R2 level and lowest BIRD-2 sensitivity [27]. Interestingly, previous studies indicated that OCI-LY-1 were more sensitive to BH3 mimetics like the non-selective Bcl-2/Bcl-XL inhibitor ABT-737 [30] and the selective Bcl-2 inhibitor venetoclax [11] than SU-DHL-4. Yet, a more detailed analysis directly comparing and correlating the response of a larger set of different Bcl-2-dependent DLBCL cancer cells to BIRD-2 *versus* venetoclax has not been performed.

## RESULTS

### Heterogeneous responses in DLBCL cell lines towards venetoclax treatment

**Figure 1:**
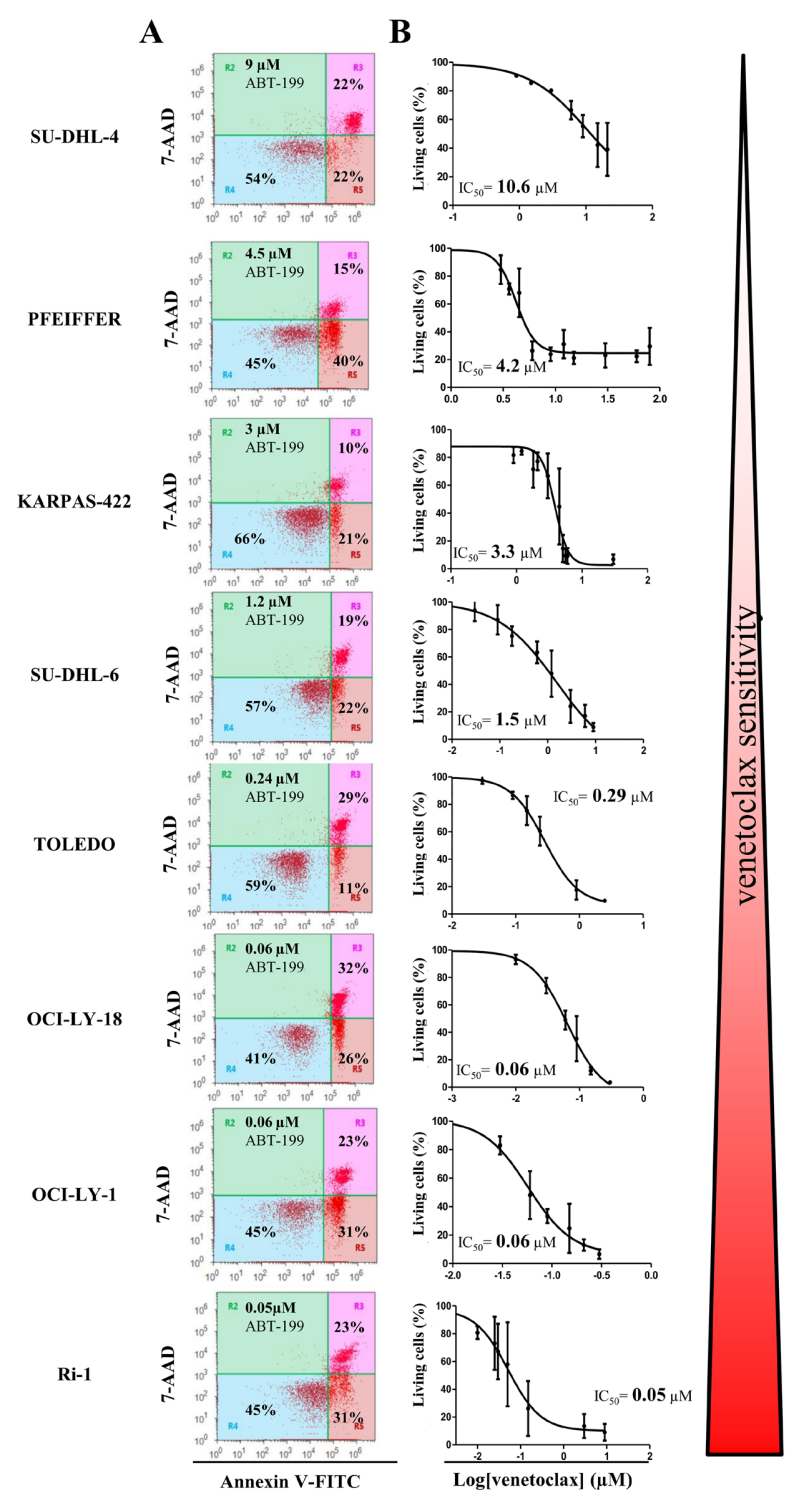
The apoptotic response of eight different DLBCL cell lines towards venetoclax treatment **(A)** Representative dot plots from flow cytometric analysis of Annexin V-FITC/7-AAD stained SU-DHL-4, PFEIFFER, KARPAS-422, SU-DHL-6, TOLEDO, OCI-LY-18, OCI-LY-1, and Ri-1 cells, treated with venetoclax at a concentration (indicated in the left top corner of the dot plot) around its IC_50_ value during 24h (10 000 cells per analysis). **(B)** Concentration-response curves of the 8 different DLBCL cell lines after incubation with increasing concentrations of venetoclax for 24h. The apoptotic population was defined as the Annexin V-FITC/7-AAD-positive fraction. Data represented are average ± SD (N≥3).

**Figure 2:**
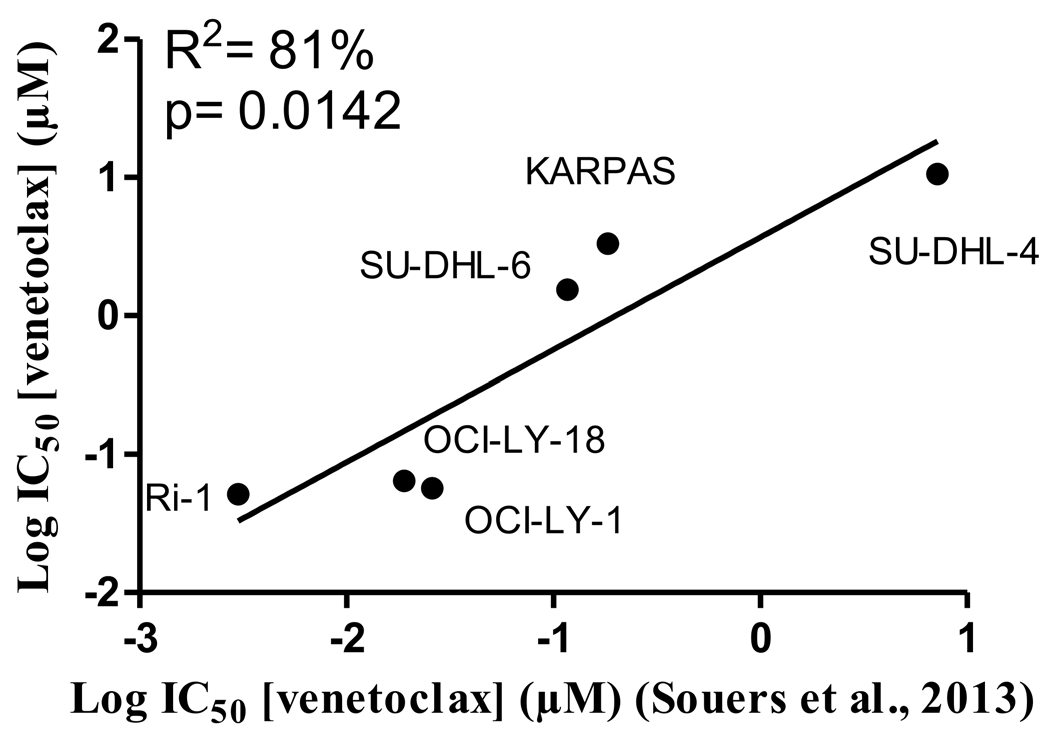
Positive correlation between the IC_50_ values for venetoclax determined in this project and previously published IC_50_ values Linear regression analysis of the IC_50_ values obtained for venetoclax from the concentration-response curves of Figure 1 against the previously published results obtained by Souers et al. [11] for respectively six different DLBCL cell lines.

A collection of cancer cell lines mainly composed of germinal center DLBCL cells, which are highly dependent on Bcl-2 to survive the continuous and permanent death signaling, was used in the present study. Although, all the cells displayed high levels of Bcl-2 and were identified to be dependent on Bcl-2 for their survival [30], they differently responded to ABT-199 (venetoclax) treatment [11]. We wanted to validate the differential apoptotic sensitivity towards venetoclax in our collection of hematological cancer cell lines. To challenge our findings, we also included an internal (negative) control, i.e. a DLBCL cell line (PFEIFFER) that was not dependent on Bcl-2, but expresses high levels of Bfl-1 mRNA and therefore was described as being putatively Bfl-1 dependent [30]. Hence, we exposed the cells to increasing concentrations of venetoclax and determined the apoptosis fraction after 24 hours of venetoclax treatment (Figure 1A and 1B). The IC_50_ was determined, confirming the differential apoptotic sensitivities in these cell lines, listed from high to low sensitivity to venetoclax: Ri-1 (IC_50_= 0.05 μM), OCI-LY-1 (IC_50_= 0.06 μM), OCI-LY-18 (IC_50_= 0.06 μM), TOLEDO (IC_50_= 0.29 μM), SU-DHL-6 (IC_50_= 1.5 μM), KARPAS-422 (IC_50_= 3.3 μM), PFEIFFER (IC_50_= 4.2 μM) and SU-DHL-4 (IC_50_= 10.6 μM). Further, we wanted to validate our data set against the results obtained by Souers et al. [11]. These data revealed, using linear regression analysis, a strong and significant positive correlation (R^2^= 81%, Figure 2) between our experimentally obtained IC_50_ values and their IC_50_ values [11]. Hence, we could confirm and validate the heterogeneity and representativeness of our cell lines towards venetoclax.

### BIRD-2 sensitivity negatively correlated with venetoclax-induced apoptosis in DLBCL cells

**Figure 3:**
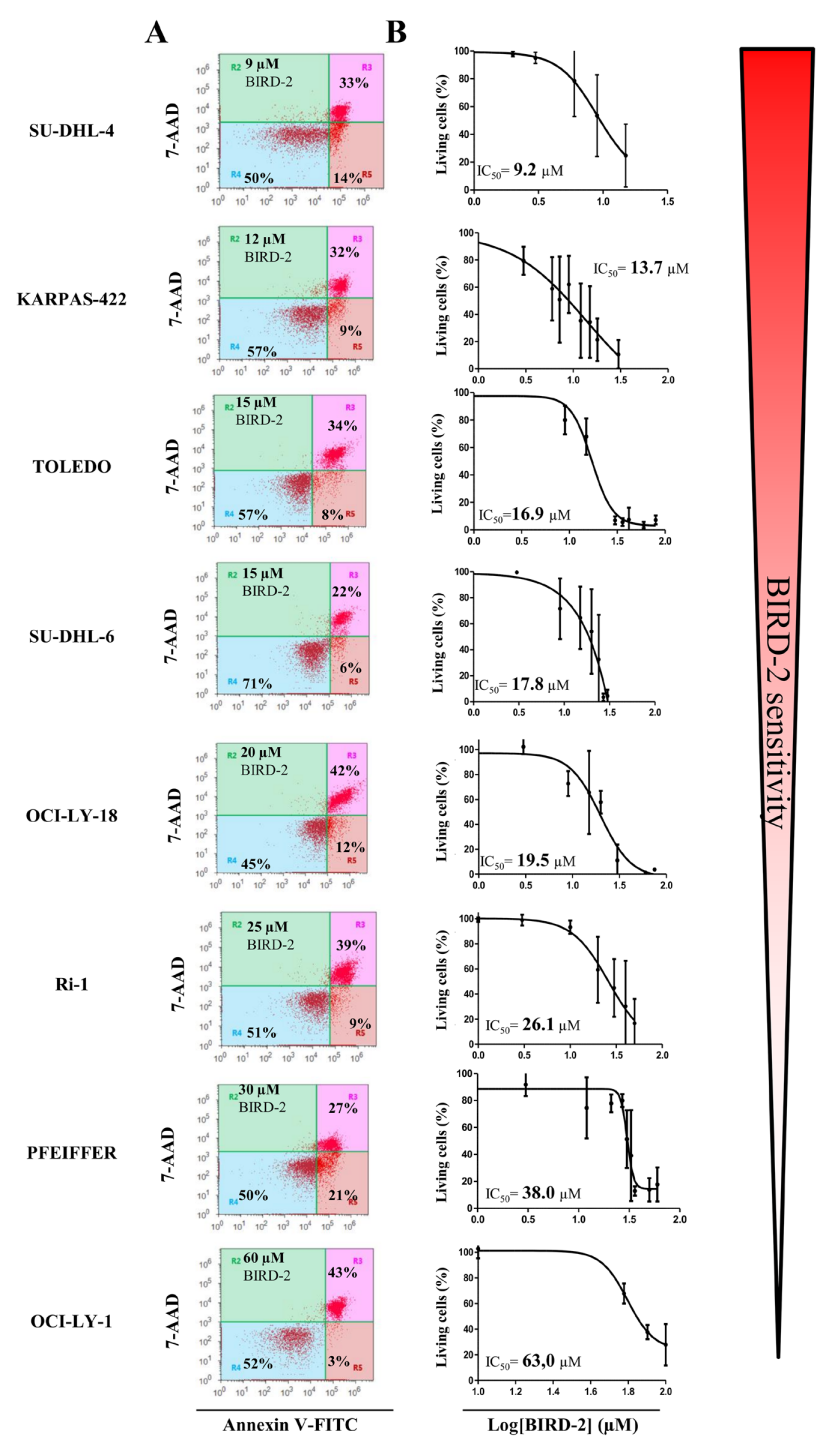
Heterogeneity in the apoptotic response of eight different DLBCL cell lines towards BIRD-2 treatment **(A)** Representative dot plots from flow cytometric analysis of Annexin V-FITC/7-AAD-stained SU-DHL-4, KARPAS-422, SU-DHL-6, TOLEDO, OCI-LY-18, Ri-1, PFEIFFER and OCI-LY-1 cells, treated with BIRD-2 at a concentration (indicated in the left top corner of the dot plot) around its IC_50_ value, during 24h (10 000 cells per analysis). **(B)** Concentration-response curves of the different DLBCL cell lines after incubation with increasing concentrations of BIRD-2 for 24h. The apoptotic population was identified as the Annexin V-FITC/7-AAD-positive fraction. Data represented are average ± SD (N≥3).

**Figure 4:**
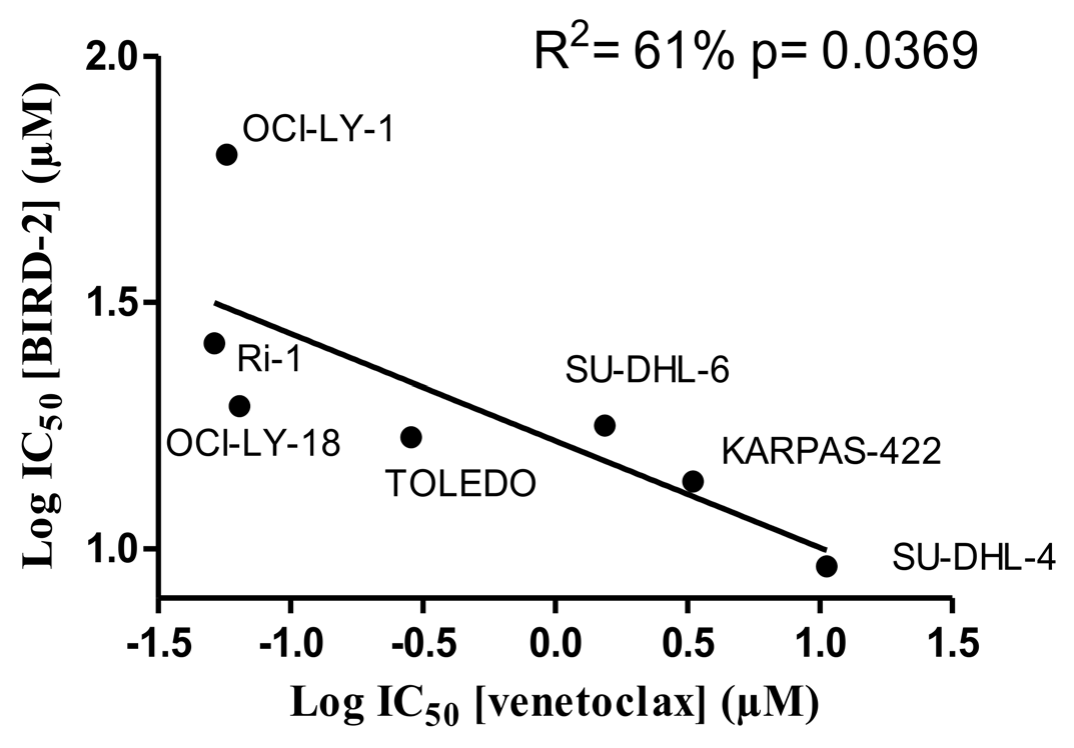
The IC_50_ values of the analysed DLBCL cell lines indicate an opposite response to BIRD-2 versus venetoclax The different IC_50_ values were determined via the concentration-response curves (Figures 1 and 3) and the log IC_50_ values for BIRD-2 were plotted as a function of the log IC_50_ values for venetoclax. These IC_50_ values were subjected to statistical analysis via linear regression.

Since BIRD-2 and venetoclax target different domains of Bcl-2, i.e. the BH4 domain and the hydrophobic cleft, respectively, we next examined the sensitivity of these cells to BIRD-2. We therefore determined the IC_50_ values for BIRD-2-induced cell death (in the following rank order from high to low sensitivity to BIRD-2: SU-DHL-4 (IC_50_= 9.2 μM), KARPAS-422 (IC_50_= 13.7 μM), TOLEDO (IC_50_= 16.9 μM), SU-DHL-6 (IC_50_= 17.8 μM), OCI-LY-18 (IC_50_= 19.5 μM), Ri-1 (IC_50_= 26.1 μM), PFEIFFER (IC_50_= 38.0 μM) and OCI-LY-1 (IC_50_= 63 μM)) and correlated them with the IC_50_ values obtained for venetoclax-induced cell death. Interestingly, cells previously shown to be sensitive towards venetoclax (Ri-1, OCI-LY-1 and OCI-LY-18, Figure 1) were more resistant towards BIRD-2 treatment (Figure 3A and 3B) and *vice versa*. Moreover, plotting the log IC_50_ values of both compounds against each other for the Bcl-2-dependent cell lines revealed an opposite sensitivity between BIRD-2 and venetoclax-induced cell death (Figure 4). The correlation between BIRD-2 and venetoclax sensitivity was quantified by linear regression, showing a significant reciprocal correlation between BIRD-2 versus venetoclax sensitivity with a R^2^ value of 61%. Strikingly, the IC_50_ values for BIRD-2 and venetoclax in the Bfl-1-dependent cell line PFEIFFER did not follow this reciprocal sensitivity. This is consistent that the opposite correlation between both Bcl-2 inhibitors is valid for Bcl-2-dependent cancer cells, but not for cancer cells dependent on other Bcl-2-family members, like Bfl-1. This underpins the “on-target” effect of BIRD-2 and venetoclax tools.

### IP_3_R2 protein expression levels positively correlated with BIRD-2 sensitivity

**Figure 5:**
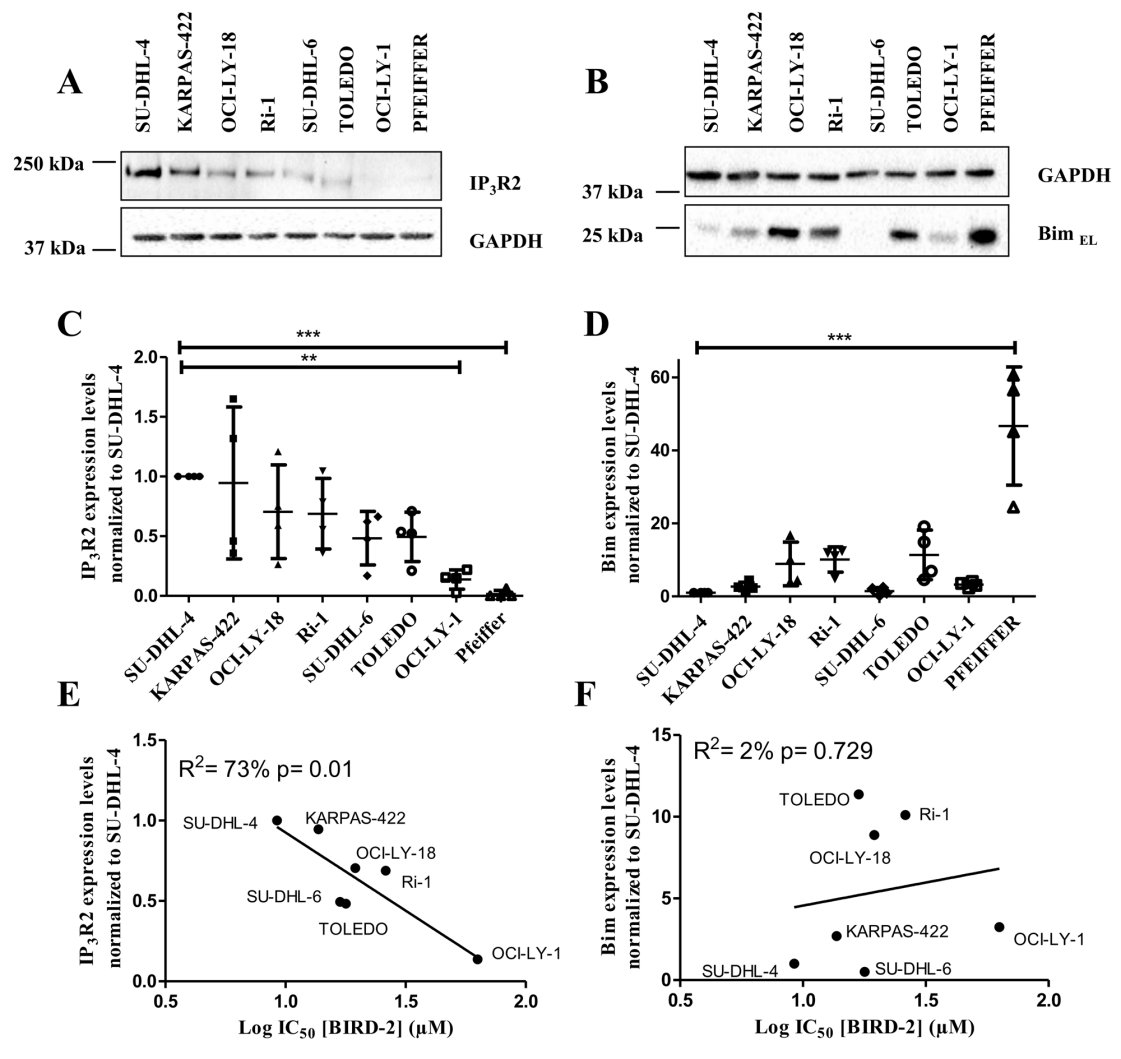
Positive correlation between the IP_3_R2 levels and the BIRD-2 sensitivity **(A-B)** Representative western blots of the IP_3_R2 (A) and Bim_EL_ (B) expression levels. GAPDH was used as loading control. **(C-D)** Quantification of IP_3_R2 (C) and Bim_EL_ (D) expression levels. Data are represented as the average ± SD of N≥3 with ^**^ p<0.01 and ^***^p<0.001 obtained via a repeated measure ANOVA with a Bonferroni’s post-hoc test versus SU-DHL-4. **(E-F)** Correlation between the expression levels of IP_3_R2 (E) and Bim_EL_ (F) with the IC_50_ values of BIRD-2, subjected to statistical analysis via linear regression.

Previously, our laboratory has provided evidence for the existence of a correlation between the BIRD-2-induced cell death and the IP_3_R2 expression levels [27]. We thereby showed that there was a good correlation with IP_3_R2, the IP_3_R isoform with the highest sensitivity for the endogenous ligand IP_3_ [29], but not with the other isoforms nor with the total IP_3_R level, pointing to a peculiar role of this IP_3_R2 isoform in cytotoxic Ca^2+^signaling. We also wanted to examine whether there was a correlation between the sensitivity towards BIRD-2 and the expression levels of the BH3-only protein Bim, which is active at the level of the mitochondria. Therefore, we quantified the expression levels of IP_3_R2 and Bim_EL_, the most abundant isoform of Bim, in DLBCL cell lysates using immunoblotting (normalized to SU-DHL-4, Figure 5A-5D). These expression levels were plotted with respect to the IC_50_ values obtained for BIRD-2 for each cell line (Figure 5E-5F). These data revealed a significant positive correlation between the BIRD-2 sensitivity and IP_3_R2- expression levels (R^2^= 73% Figure 5E), but no correlation between the Bim-expression levels and BIRD-2 sensitivity was found (R^2^= 2%, Figure 5F). As a consequence, SU-DHL-4, KARPAS-422 and TOLEDO cells were good responders towards BIRD-2 (Figure 5E) due to their high IP_3_R2 expression levels (Figure 5C). On the other hand, OCI-LY-1 has a lower amount of IP_3_R2 (Figure 5C), which correlates with the low sensitivity to BIRD-2 (Figure 5E). Also, PFEIFFER expressed lower IP_3_R2 levels but its position in the plot showed a bad correlation with BIRD-2 sensitivity (data not shown).

### Intracellular Ca^2+^ is required for BIRD-2-, but not for venetoclax-induced cell death

**Figure 6:**
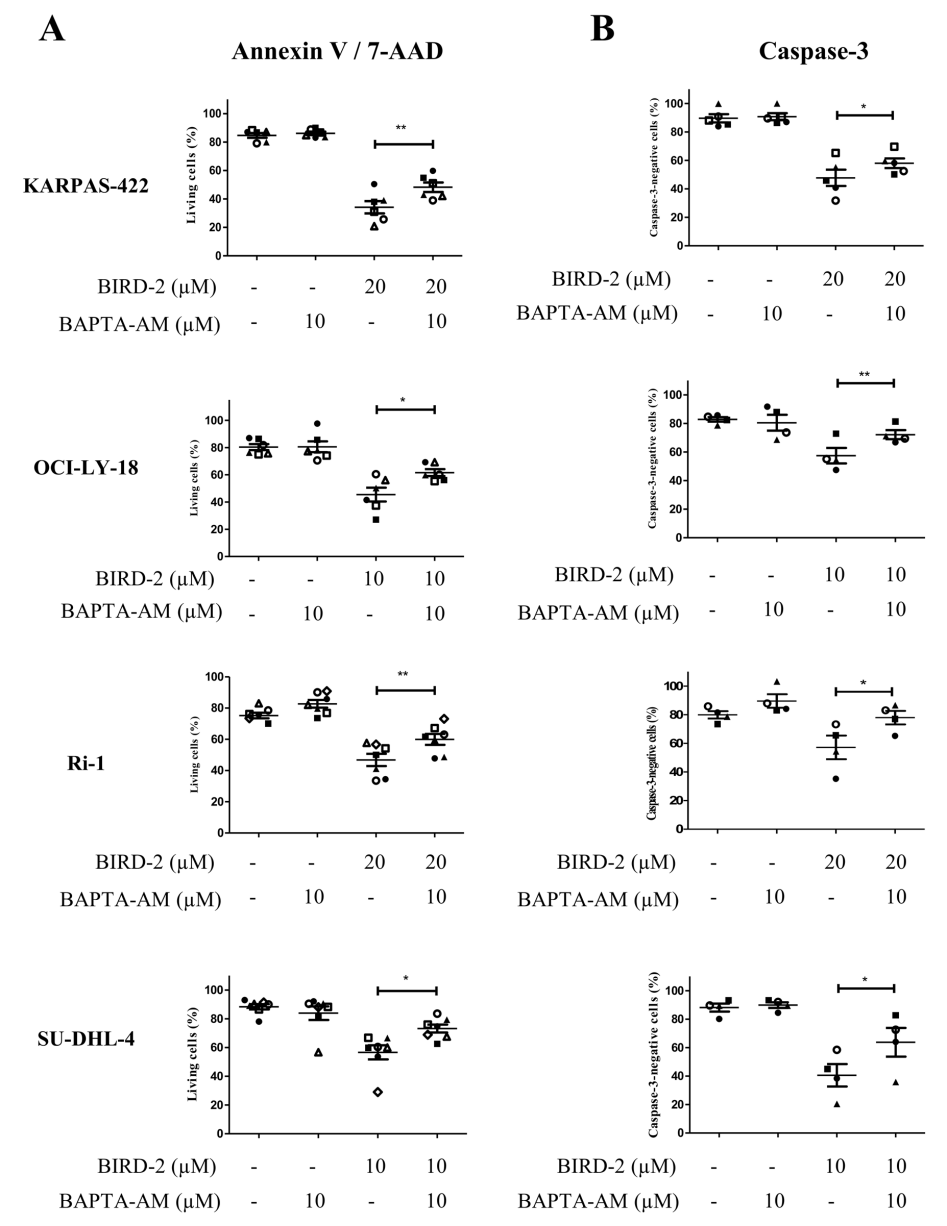
Intracellular Ca^2+^ has a primary role in the BIRD-2-induced cell death Analysis of Annexin V-FITC/7-AAD-negative cells (living cells (%), **A**) and caspase-3-negative cells **(B)** obtained using flow-cytometric analysis of KARPAS-422, OCI-LY-18, Ri-1 and SU-DHL-4 cells treated with or without BIRD-2 and 10 μM BAPTA-AM for 2h. Data are represented as mean ± SEM >3 independent experiments. Significance was obtained using a two-tailed paired *t*-test with ^*^ p< 0.05, ^**^p<0.01.

**Figure 7:**
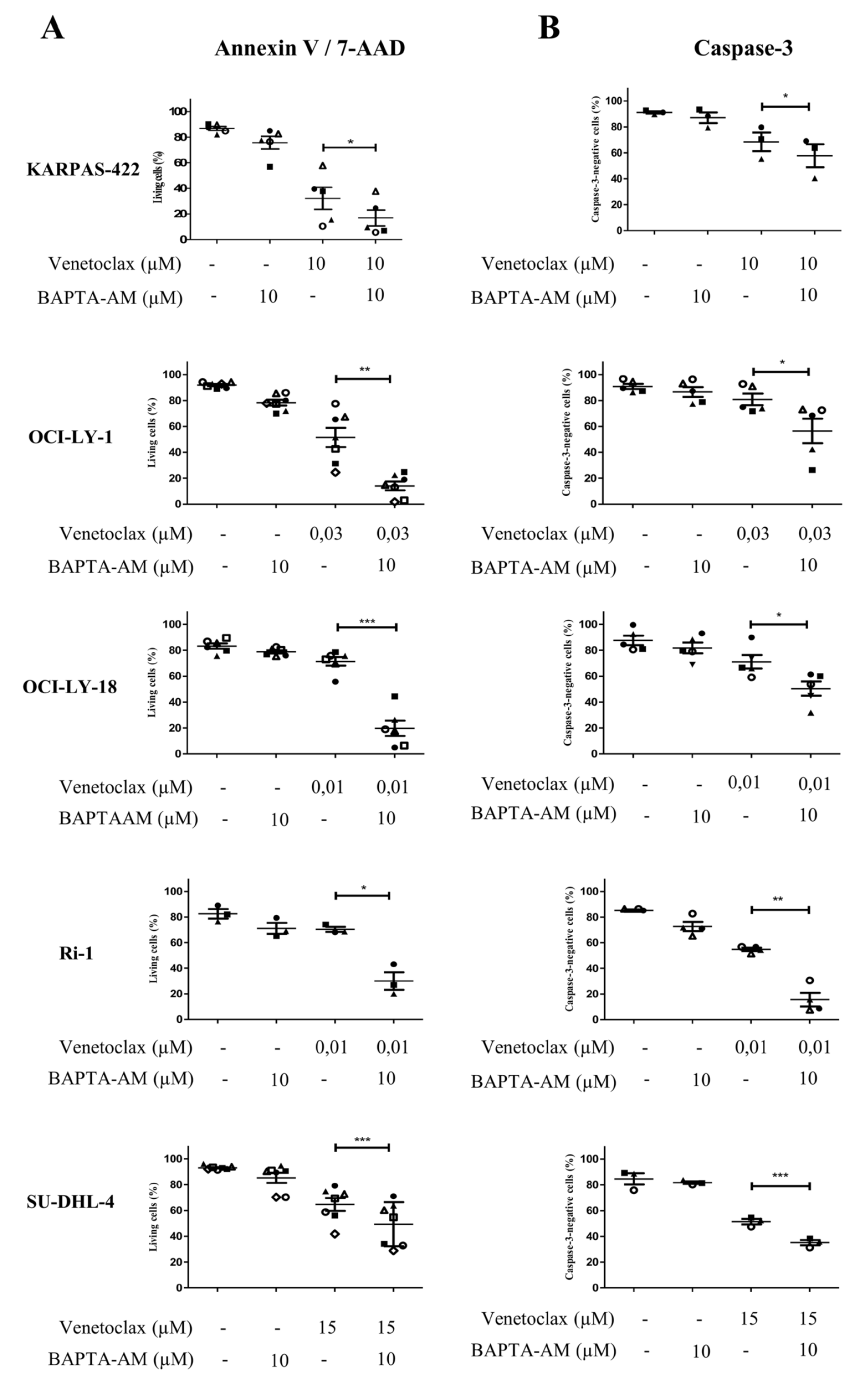
Intracellular Ca^2+^ does not contribute to the venetoclax-induced cell death Analysis of Annexin V-FITC/7-AAD-negative cells (living cells (%), **A**) and caspase-3-negative cells **(B)** obtained using flow-cytometric analysis of KARPAS-422, OCI-LY-1, OCI-LY-18, Ri-1 and SU-DHL-4 cells treated with or without venetoclax and 10 μM BAPTA-AM for 4h. Data are represented as mean ± SEM >3 independent experiments. Significance was obtained using a two-tailed paired *t*-test with ^*^ p< 0.05, ^**^p<0.01, ^***^p<0.001.

In a previous study, we have already observed that the BIRD-2-induced apoptosis correlated with the BIRD-2-induced Ca^2+^ release [27]. This together with the observation that BIRD-2 positively correlates with the IP_3_R2-expression levels strongly suggest that intracellular Ca^2+^ is important for the BIRD-2-induced cell death. To explore the role of Ca^2+^ in BIRD-2- and venetoclax-induced cell death, we measured apoptosis while chelating intracellular Ca^2+^ using BAPTA-AM. We first validated that our BAPTA-AM treatment effectively chelated intracellular Ca^2+^ by assessing agonist-induced Ca^2+^ release in Fura-2-loaded SU-DHL-4 cells. Compared to untreated cells, cells pre-treated with BAPTA-AM for 2 hours displayed a severely reduced response to anti-IgG/IgM, a B-cell receptor agonist that results in IP_3_/Ca^2+^ signaling, thereby validating the effectiveness of BAPTA-AM (Supplementary Figure 1). Adding BAPTA-AM prior to BIRD-2 treatment significantly reduced the occurrence of cell death in KARPAS-422, OCI-LY-18, Ri-1 and SU-DHL-4 (Figure 6A). A similar protection by BAPTA-AM was observed when measuring apoptosis via a caspase-3 assay (Figure 6B). OCI-LY-1 could not be included in this analysis as the concentration of BIRD-2 needed to evoke cell death was too high to exclude additional off-target effects. Oppositely, chelating intracellular Ca^2+^ upon venetoclax treatment significantly enhanced single-agent induced cell death and caspase-3 activity in KARPAS-422, OCI-LY-1, OCI-LY-18, SU-DHL-4 and Ri-1 cells (Figure 7A and 7B). Interestingly, the cell death induced by BAPTA-AM in the presence of venetoclax seemed to correlate with their venetoclax sensitivity, as Ri-1, OCI-LY-1 and OCI-LY-18 were the most sensitive to BAPTA-AM and KARPAS-422 and SU-DHL-4 the least sensitive ones.

Hence, overall these findings suggest a different requirement for Ca^2+^ in the BIRD-2- or venetoclax-induced cell death.

### Synergistic killing of DLBCL cells by venetoclax and BIRD-2

**Figure 8:**
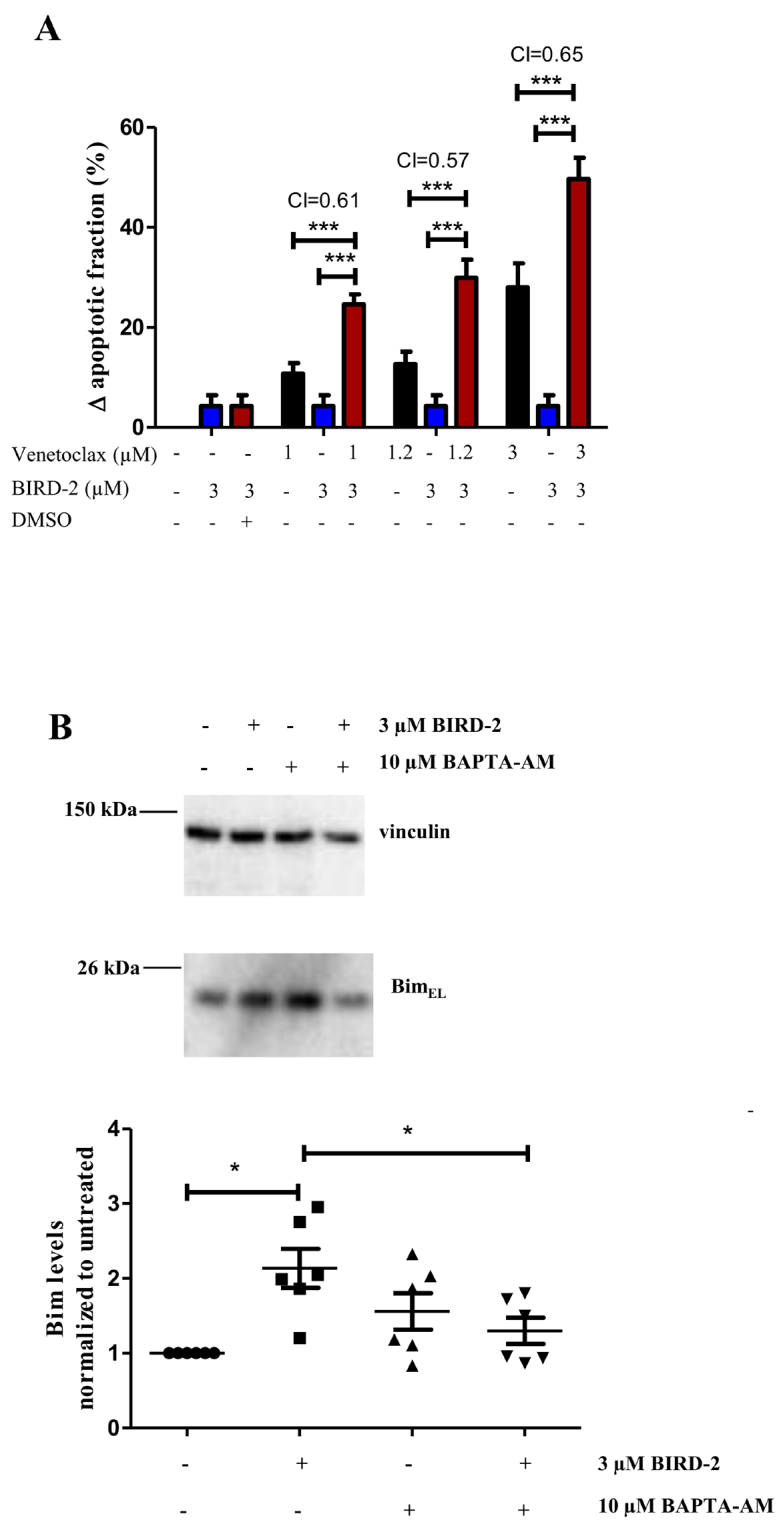
Synergistic effect of BIRD-2 and venetoclax in SU-DHL-4 cells, correlating to Bim upregulation in BIRD-2-treated SU-DHL-4 cells **(A)** SU-DHL-4 cells were treated for 24h with various concentrations of venetoclax alone (black bars), BIRD-2 alone (blue bars) or a combination of venetoclax/DMSO Ctrl (0.03%) and BIRD-2 (red bars). Cell death was measured using flow cytometry of Annexin V-FITC/7-AAD-stained cells and plotted as the BIRD-2- or venetoclax-induced apoptotic fraction. The combination index, calculated using the response additive method (CI= (E_venetoclax_+ E_BIRD-2_)/E_venetoclax+BIRD-2_) was measured as indication for synergistic or additive cell killing. Data are represented as average ± SEM of N=5. Statistical significance was determined with a two-way ANOVA with a Bonferroni post-hoc test comparing E_venetoclax_ or E_BIRD-2_ with E_venetoclax+BIRD-2_ for the different venetoclax concentrations. **(B)** Representative western blots showing the Bim_EL_-expression levels after 24h treatment of SU-DHL-4 cells with 3 μM BIRD-2 in the presence or absence of 10 μM BAPTA-AM. Vinculin was used as loading control. Data are represented as the average ± SD of N≥3 with ^*^ p<0.05 obtained via Wilcoxon signed rank test.

Because BIRD-2 and venetoclax target different domains of Bcl-2, we tested whether combining these agents would synergistically induce cell death. Combining a submaximal concentration of BIRD-2 with increasing concentrations of venetoclax enhanced cell-death induction in the venetoclax-resistant SU-DHL-4 DLBCL cell line compared to single-agent treatment (Figure 8A). In order to determine mathematically whether it is a synergistic or additive effect, a combination index (CI) was measured. As a CI<1 indicates synergy, co-treatment of SU-DHL-4 with BIRD-2 and venetoclax consistently induced synergistic cytotoxicity at all concentration measured. Additionally, similar results were obtained when calculating the CI using the raw data set (Supplementary Figure 2) instead of the change in apoptotic fraction. Moreover, we demonstrated that treating the highly BIRD-2-sensitive SU-DHL-4 cell line with 3 μM BIRD-2, a concentration ineffective to trigger cell death by itself, significantly increased the Bim levels, thereby priming the cells for sensitivity to venetoclax (Figure 8B). Moreover, the BIRD-2-induced upregulation of Bim seemed to be Ca^2+^ dependent, since chelating intracellular Ca^2+^ with BAPTA-AM could suppress the BIRD-2-induced upregulation of Bim.

Together with the existence of a reciprocal correlation, these data underpin differences in the targets for apoptotic induction by BIRD-2 or venetoclax.

## DISCUSSION

Anti-apoptotic Bcl-2 protects cancer cells from cell death by acting at the mitochondria, scaffolding Bim/Bak/Bax [6] and by acting at the ER, suppressing excessive IP_3_Rs activity [31]. Here, we used a collection of well-established Bcl-2-dependent DLBCL cell lines to assess the cell-death properties of two selective Bcl-2 inhibitors that target different domains of Bcl-2, namely ABT-199 (venetoclax), which targets the hydrophobic cleft of Bcl-2 and disrupts Bcl-2/Bim interactions [11], and BIRD-2, which targets the N-terminal BH4 domain of Bcl-2 and disrupts IP_3_R/Bcl-2 interactions [21]. Plotting the IC_50_ values for the apoptotic effect of both Bcl-2 inhibitors revealed a reciprocal sensitivity between venetoclax and BIRD-2 in the cancer cells dependent on Bcl-2 for their survival. This reciprocal sensitivity between venetoclax and BIRD-2 appeared to count for Bcl-2-dependent cancer cells, but not for Bfl-1-dependent cancer cells, as the Bfl-1-dependent cancer cell line PFEIFFER was relatively resistant to both venetoclax and BIRD-2. This correlates with previous work showing that increased transcript levels of Bfl-1 has been allocated to resistance to BH3 mimetics [32]. We also wish to note that the range of sensitivities of the different DLBCL cells obtained for BIRD-2 versus venetoclax are very different, being ∼6-fold different for BIRD-2, while being ∼200-fold different for venetoclax. The reason for this difference is not clear, but may relate to differences in uptake rate and mechanisms and in their underlying mechanism of action. In any case, the range of sensitivities obtained in this work for BIRD-2 and venetoclax are very similar to the ones previously reported in the literature [11, 27, 28, 33].

Though our study is the first to have systematically compared and correlated the sensitivity of a collection of DLBCL cell lines towards venetoclax versus BIRD-2 by performing complete and quantitative concentration-response analyses, previous studies have hinted towards such a concept. Indeed, previous work from our own lab focusing on SU-DHL-4 versus OCI-LY-1 and short-term apoptosis experiments showed that the venetoclax-sensitive OCI-LY-1 cells were resistant to 10 μM BIRD-2, while SU-DHL-4, a cell line less sensitive to venetoclax was sensitive to 10 μM BIRD-2 [27]. Follow-up work by Distelhorst and co-workers in multiple myeloma cells [28] and small cell lung carcinoma [34], using a single concentration of BIRD-2 and of BH3 mimetics, confirmed that cell lines more resistant to BH3 mimetics were more sensitive to BIRD-2 and *vice versa*. We now provide further insight in the reciprocal sensitivity of cancer cells to venetoclax versus BIRD-2. Previous work from Letai and co-workers showed that responsiveness to BH3 mimetics correlated with Bim levels and more particular with Bcl-2/Bim complex formation [30]. Here, we show that BIRD-2 sensitivity positively correlates with IP_3_R2 levels while there was no correlation with Bim levels. Hence, these data support the concept of the dual dependence of cancer cells on Bcl-2 for their survival with respect to the oncogenic signaling either by Bim upregulation or by IP_3_R2 upregulation. Of note, Bcl-2 also interacts with the regulatory and coupling domain of the IP_3_R isoform 1 and isoform 3 [22] and is able to form protein complexes with all three IP_3_R isoforms in Jurkat and WEHI7.2 T cells [18, 35]. This correlates with the high level of conservation of the 20-amino acid stretch encompassing the Bcl-2-binding site among the different IP_3_R isoforms [36–38]. However, in DLBCL cells, it seems that Bcl-2 interaction with the different IP_3_R isoforms is context dependent. In SU-DHL-4, Bcl-2 co-immunoprecipitated predominantly with IP_3_R2 when compared with IP_3_R3, while in OCI-LY-1 the reverse was observed [27]. The reason for this is not clear, but may indicate a differential modulation of IP_3_R isoforms and Bcl-2 in different cellular contexts. In any case, both the intracellular Ca^2+^ release and the apoptotic response induced by BIRD-2 correlated with IP_3_R2-expression levels but not with IP_3_R1- or IP_3_R3-expression levels. The binding of Bcl-2 to IP_3_R2, which displays the highest sensitivity to its ligand IP_3_, and the concomitant suppression of IP_3_R2 activity may enable cancer cell survival in two ways: (i) by preventing the occurrence of excessive, pro-apoptotic Ca^2+^-release events and (ii) by establishing low-level Ca^2+^ signaling that drives the mitochondrial metabolism of cancer cells [39, 40]. In addition to this, IP_3_R2 upregulation has been implicated in cellular senescence, a stable growth arrest that prevents the proliferation of malignant cells [41]. Therefore, the binding of Bcl-2 to IP_3_R2 may allow cancer cells to escape cellular senescence. The oncogenic mechanisms responsible for IP_3_R2 upregulation remain largely unknown, although a role for constitutive NFAT signaling may be part of the signaling mechanisms that cause IP_3_R2 upregulation [42]. Furthermore, it is also unknown which mechanisms or checkpoints are in place that are responsible for the ‘choice’ of upregulating Bim versus IP_3_Rs in response to oncogenic stress. It is conceivable that the upregulation of Bim or IP_3_R2 in cancer cells ought to be compensated by high anti-apoptotic Bcl-2 levels resulting in protein complexes with either Bim or IP_3_R2. Because the IP_3_R2 is an important mediator of the BIRD-2 susceptibility, we examined in more detail the role of intracellular Ca^2+^ in the BIRD-2- and venetoclax-induced cell death. We confirmed earlier work [27, 28], pinpointing to an important role for intracellular Ca^2+^ overload in BIRD-2-induced cell death, since intracellular Ca^2+^ buffering decreased caspase-3 activation in response to BIRD-2 treatment in different DLBCL cell lines. In addition to this, it is possible that BAPTA-AM can suppress BIRD-2-induced cell death by counteracting the upregulation of pro-apoptotic BH3-only protein Bim brought about by BIRD-2 treatment. In contrast, chelating intracellular Ca^2+^ enhanced the venetoclax-induced caspase-3 activation in different DLBCL cell lines. We have already elucidated previously that venetoclax did not trigger an acute Ca^2+^ release and that intracellular Ca^2+^ buffers did not protect against venetoclax-induced cell death in OCI-LY-1 and SU-DHL-4 cells [25]. Here we extended our earlier work to several DLBCL cell lines, firmly excluding an essential role for intracellular Ca^2+^ overload for venetoclax-induced cell death. On the contrary, it seems that DLBCL cells can be sensitized to venetoclax by buffering intracellular Ca^2+^. The mechanisms underlying the interplay between venetoclax and basal Ca^2+^ signals in DLBCL are not clear and will require further research. However, it is possible that BAPTA-AM treatment disturbs the balance between pro-apoptotic and anti-apoptotic Bcl-2-family members. For instance, ovarian cancer cells exposed to BAPTA-AM display a reduction in the protein levels of anti-apoptotic Mcl-1 [43]. Hence, such decreases in the levels of anti-apoptotic proteins and/or increases in the levels of pro-apoptotic proteins may account for the toxic effects of BAPTA-AM and/or sensitization towards BH3-mimetic drugs.

In addition, combing BIRD-2 with venetoclax enhanced cell demise compared to single treatment alone in a venetoclax-resistant DLBCL cell line. Hence, co-treatment with BIRD-2 could sensitize the cells towards venetoclax. Previous reports also indicated a synergistic effect between BIRD-2 and BH3 mimetics in melanoma [28] and lung cancer cells [34]. In melanoma cells prolonged exposure to BIRD-2 increased the protein levels of Bim in a Ca^2+^-dependent manner and consequently increased the sensitivity towards BH3 mimetics [28]. We here report that the BIRD-2-dependent upregulation of Bim also occurs in DLBCL cells in a Ca^2+^-dependent manner, thereby sensitizing these cancer cells towards venetoclax and thus likely underlying the synergism between BIRD-2 and venetoclax for inducing cell death in DLBCL cells. This synergism also establishes an interesting link between the “ER Ca^2+^ signaling” pathways and “mitochondrial BH3-only” pathways in apoptosis, where Ca^2+^ signals may not only by themselves trigger apoptosis, e.g. by mitochondrial Ca^2+^ overload, but may also affect the expression levels of other Bcl-2-family members, thereby modulating cell death. This intriguing link between Ca^2+^ signaling and Bcl-2-family members is further underpinned by the fact that venetoclax-induced cell death can be enhanced by intracellular Ca^2+^ chelation using BAPTA-AM.

Our collection of data is also consistent with the dual role of anti-apoptotic Bcl-2 being either inhibiting pro-apoptotic proteins or suppressing aberrant IP_3_-mediated Ca^2+^ release [31]. It may also indicate that different domains of Bcl-2 underlie Bcl-2’s differential role at the mitochondria (its hydrophobic cleft targetable by BH3-domain-like molecules) and at the ER (its BH4 domain targetable by IP_3_R-domain-derived molecules). Indeed, previous work indicated that the inhibition of IP_3_Rs by Bcl-2 critically depends on Bcl-2’s BH4 domain [21, 22], while Bcl-2’s hydrophobic cleft is dispensable for this [24]. Furthermore, by showing that the IP_3_R2 protein levels correlated with the sensitivity to BIRD-2 in the following rank order: KARPAS-422 > SU-DHL-4 > OCI-LY-18 > Ri-1 > SU-DHL-6 > TOLEDO > OCI-LY-1, we confirm the complex formation between IP_3_R2 and Bcl-2 as a major determinant for cancer cell addiction to Bcl-2 at the ER. However, other confounding factors might further influence the correlation between the IP_3_R2/Bim-expression levels with the BIRD-2 sensitivity. Indeed, recent reports have indicated that the activation of proto-oncogene like PKB/Akt can affect, by phosphorylation, both the IP_3_R function and ER Ca^2+^ homeostasis and thereby decreasing apoptotic events by dampening the mitochondrial Ca^2+^ rise [44, 45]. Moreover, this oncogenic PKB/Akt hyperactivity is often linked to the loss of PTEN in DLBCL cells [46]. It is not known whether PKB/Akt signaling affects all IP_3_R subtypes and particularly IP_3_R2 in the same way and thus could modulate BIRD-2-induced cell death.

Thus, cancer cells can exploit both of the anti-apoptotic actions of Bcl-2 to promote their survival, dependent on the challenging conditions operative in these cells, either by Bim upregulation or by IP_3_R2 upregulation. The type of addiction to Bcl-2 will dictate their apoptotic sensitivity to BH3 mimetics or BH4-domain-targeting compounds. Importantly, the chemotherapeutic response of cancer cells appears to correlate with the mitochondrial “primed to death” status of Bcl-2 and thus their sensitivity to BH3 mimetics [14, 47–49]. This indicates that patients displaying a poor response to chemotherapy will also display a weak response to BH3 mimetics, as they impinge on the same mitochondrial pathway. However, our data suggest that these “resistant” cancers might be targeted using BH4-domain-targeting tools like BIRD-2. This indicates that patients with a poor clinical response to chemotherapy due to poor mitochondrial priming may benefit from BH4-domain-antagonizing compounds, like BIRD-2. Further work is needed on the identification of BH4-domain-antagonizing molecules that are applicable *in vivo*. However, this area is also in vivid expansion, recently resulting in the development of BDA-366, a small molecule targeting the BH4 domain of Bcl-2 with very high affinity and selectivity compared to Bcl-XL [50]. The mechanism of action of BDA-366 appeared to involve a conformational switch of Bcl-2 from an anti-apoptotic to pro-apoptotic protein, thereby exposing its BH3 domain and acting as a Bax-activating protein. BDA-366 also disrupted IP_3_R/Bcl-2 complexes [51]. Yet, further work is required to validate whether BIRD-2 and BDA-366 share a common mechanism of action. As such, BDA-366 might be a first promising tool to elicit cell death in cancers that are more resistant to BH3 mimetics [50–52].

Overall, our data indicate that BH4-domain-antagonizing tools like BIRD-2 hold potential to kill Bcl-2-dependent cancer cells, in particular those that are more resistant to chemotherapy and precision medicines like venetoclax. By acting on a different functional domain of Bcl-2 than BH3 mimetics, these tools could provide additional opportunities to kill these cancers, and to be used as follow-up strategies to kill venetoclax-resistant or -relapsed cancers, or to participate in combination therapies with venetoclax via their ability to boost endogenous Bim levels.

## MATERIALS AND METHODS

### Cells

SU-DHL-4, KARPAS-422, PFEIFFER, TOLEDO, SU-DHL-6, OCI-LY-1 and OCI-LY-18 DLBCL cell lines were kindly obtained from Dr. Anthony Letai (Dana-Farber Cancer Institute, Boston, Massachusetts, USA), who performed a complete “BH3 profiling” analysis of these cell lines [30]. The Ri-1 DLBCL cell line was ordered via DSMZ (Braunschweig, Germany). All these cell lines were authenticated by the University of Arizona Genetics Core (Tucson, AZ, USA) using autosomal short tandem repeat (STR) profiling via Science Exchange (www.scienceexchange.com). The results were validated using reference databases such as DSMZ (Germany) and sample profiles (allelic values) and electropherogram trace data were provided. All cell lines except one displayed a perfectly matched profile with 8 tested alleles (8/8), while for SU-DHL-6 cells 7/8 alleles matched. The apoptotic response of these cells to different venetoclax concentrations was validated and benchmarked against the published venetoclax IC_50_ values by Souers et al [11]. The SU-DHL-4, KARPAS-422, PFEIFFER, TOLEDO, Ri-1 and SU-DHL-6 DLBCL cell lines were cultured in suspension in RPMI-1640 media. The OCI-LY-1 and OCI-LY-18 DLBCL cell lines were cultured in suspension in Iscove modified Dulbecco medium (Invitrogen, Merelbeke, Belgium). All media were supplemented with 10% heat-inactivated fetal bovine serum, L-glutamine (100 × GlutaMAX, Gibco/Invitrogen, Merelbeke, Belgium) and penicillin and streptomycin (100 × Pen/Strep, Gibco/Invitrogen, Merelbeke, Belgium) and cultured at 37°C and 5% CO_2_.

### Antibodies and reagents

Immunoblotting was performed with anti-GAPDH (Sigma-Aldrich, Munich, Germany), anti-vinculin (Sigma-Aldrich, Munich, Germany), anti-IP_3_R2 (Rbt02 [53]) and anti-Bim (Bioké, Leiden, The Netherlands).

BIRD-2 (RKKRRQRRRGGNVYTEIKCNSLLPLAAIVRV) (purity>85%) was purchased from LifeTein (South Plainfield, New Jersey, USA) and venetoclax from Active Biochem (Bonn, Germany).

### Western blotting

DLBCL cells were washed with phosphate-buffered saline and incubated at 4°C with lysis buffer (20 mM Tris-HCl (pH 7.5), 150 mM NaCl, 1.5 mM MgCl_2_, 0.5 mM dithiothreitol, 1% Triton X-100, 1 tablet complete EDTA-free protease inhibitor per 50 ml (Thermo Scientific, Brussels, Belgium) for 30 min on a head-over-head rotor. Cell lysates were centrifuged for 5 minutes at 10 000 rpm. Twenty μg of protein sample was loaded on a NuPAGE 3-8% Tris-Acetate protein gel for the detection of the IP_3_R2 protein and for the detection of the Bim protein a NuPAGE 4-12% Bis-Tris protein gel (Life Technologies, Brussels, Belgium) was used for western blotting as previously described [22].

### Apoptosis assay

After treatment (see figure legends for more information about the treatment, time and concentration) DLBCL cells (5 × 10^5^ cells/ml) were pelleted by centrifugation, and incubated with Annexin V-FITC (Life Technologies, Brussels, Belgium) and 7-aminoactinomycin D (7-AAD) (Life Technologies, Brussels, Belgium) or with 2.5 μM NucviewTM 488 caspase-3 substrate (Biotium, CA, USA) for 15 or 30 minutes respectively. Cell suspensions were analyzed with an Attune^®^ Acoustic Focusing Flow Cytometer (Applied Biosystems, Brussels, Belgium). Cell death was scored by quantifying the population of Annexin V-FITC-positive and 7-AAD positive cells or by quantifying the caspase-3 positive cells.

### Cytosolic Ca^2+^ measurements in intact cell populations

SU-DHL-4 cells were loaded for 30 min with 1.25 μM Fura-2 AM at room temperature in modified Krebs solution (150 mM NaCl, 5.9 mM KCl, 1.2 mM MgCl_2_, 11.6 mM HEPES pH 7.3, 11.5 mM glucose and 1.5 mM CaCl_2_). Afterwards, 400 000 cells were seeded in poly-L-lysine coated 96-well plates (Greiner, Vilvoorde, Belgium) and incubated for at least 30 minutes in the absence of Fura-2 AM. Fluorescence was monitored on a FlexStation3 microplate reader (Molecular Devices, CA, USA) by alternately exciting the Ca^2+^ indicator at 340 and 380 nm and collecting emission fluorescence at 510 nm. After 60 seconds agonist-induced Ca^2+^ release was induced by adding 12 μg/ml anti-IgG/IgM (Sanbio, Uden, The Netherlands). All traces are shown as the ratio of emitted fluorescence of Fura-2 (F340/F380).

### Statistical analysis

Results from the western blot analysis are expressed as average ± SD whereby N refers to the number of independent experiments. Significance was determined using a repeated measures one-way ANOVA with a Bonferroni’s post hoc test for the IP_3_R and Bim expression levels. Statistical significance of the Bim expression levels after BIRD-2 treatment in the absence or presence of BAPTA-AM was performed with a Wilcoxon signed rank test. Differences were considered significant at p<0.05. Correlation of the different IC_50_ values with each other and with the protein expression levels were statistically analysed via linear regression.

As indicative of synergy between BIRD-2 and venetoclax treatment the Combination Index (CI) was calculated by making the ratio of the theoretical sum of the individual effects (E_venetoclax_+ E_BIRD-2_) with the effect of combining the treatments (E_venetoclax+BIRD-2_). Statistical significance was determined with a two-way ANOVA with a Bonferroni post-hoc test comparing E_venetoclax_ or E_BIRD-2_ with E_venetoclax+BIRD-2_ for the different venetoclax concentrations.

## SUPPLEMENTARY MATERIALS FIGURES


