# Supplementary material for: Reciprocal sensitivity of diffuse large B-cell lymphoma cells to Bcl-2 inhibitors BIRD-2 versus venetoclax
Source: Oncotarget. 2017 Dec 4;8(67):111656–71. doi: 10.18632/oncotarget.22898 (PMC5762350; doi:10.18632/oncotarget.22898)
Supplement: Supplementary file 1 [file oncotarget-08-111656-s001.pdf]

Reciprocal sensitivity of diffuse large B-cell lymphoma cells to Bcl-2 inhibitors BIRD-2 versus venetoclax

SUPPLEMENTARY MATERIALS

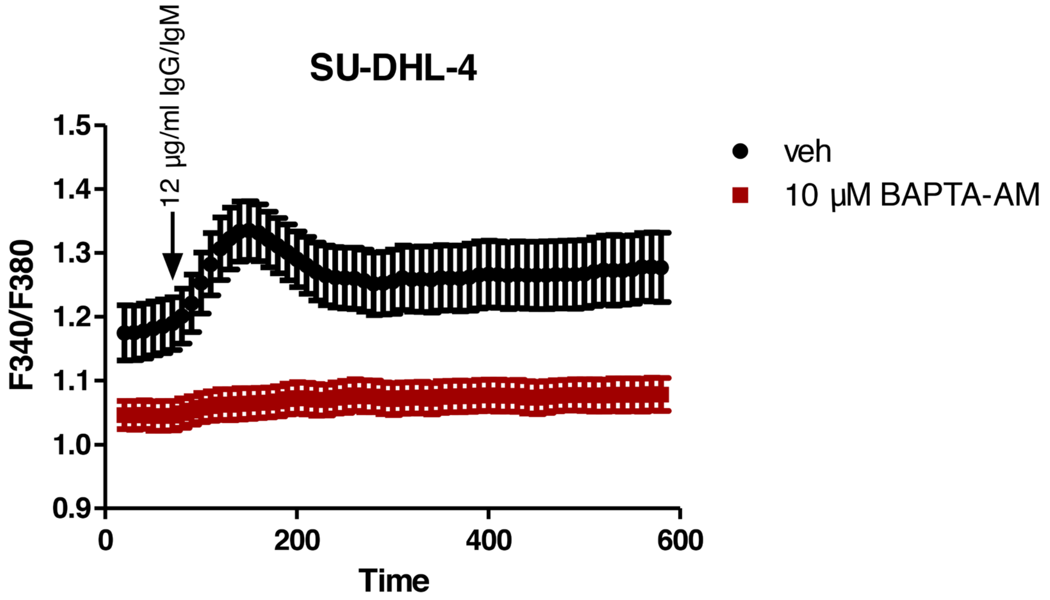

Supplementary Figure 1: BAPTA-AM treatment effectively chelates intracellular Ca^{2+} in SU-DHL-4 cells. Cytosolic Ca^{2+} measurements in Fura-2 AM-loaded SU-DHL-4 cells pretreated (red traces) or not (black traces) for 2 hours with 10 µM BAPTA-AM. Agonist-induced Ca^{2+} release was triggered via the addition of the B-cell receptor agonist (12 µg/µl anti-IgG/IgM) 60 seconds after the start of the measurement. Data represent average ± SEM of triplicate samples of 4 independent experiments.

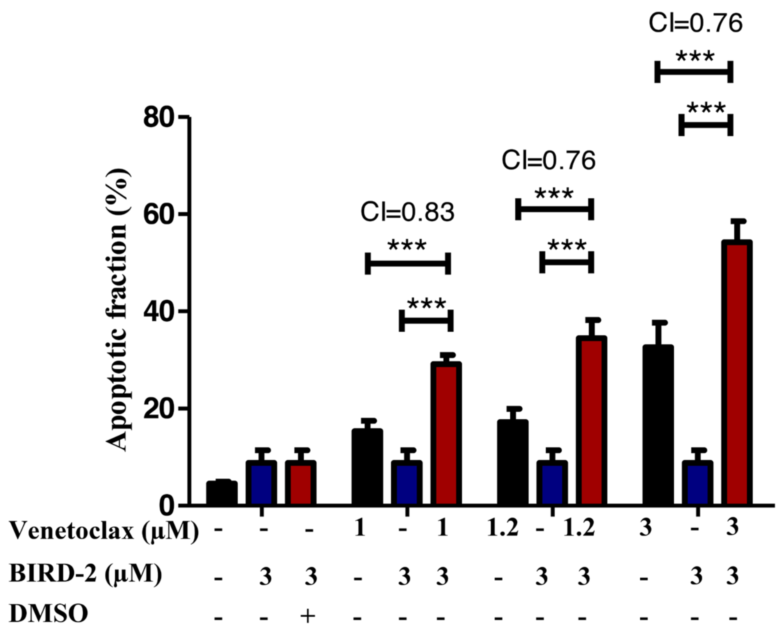

Supplementary Figure 2: Synergistic effect of BIRD-2 and venetoclax in SU-DHL-4 cells. SU-DHL-4 cells were treated for 24 hours of venetoclax alone (black bars), BIRD-2 alone (blue bars) or a combination of venetoclax/DMSO Ctrl (0.03%) and BIRD-2 (red bars). Cell death was measured using flow cytometry of Annexin V-FITC/7-AAD-stained cells and plotted as the BIRD-2- or venetoclax-induced apoptotic fraction. Similarly to Figure 8A the CI was calculated using the response additive method (CI= $(E_{venetoclax} + E_{BIRD\text{-}2})/E_{venetoclax+BIRD\text{-}2}$). Data are represented as average ± SEM of N=5. Statistical significance was determined with a two-way ANOVA with a Bonferroni post-hoc test comparing $E_{venetoclax}$ or $E_{BIRD\text{-}2}$ with $E_{venetoclax+BIRD\text{-}2}$ for the different venetoclax concentrations.
